# Non-Singleton Type-3 Fuzzy Approach for Flowmeter Fault Detection: Experimental Study in a Gas Industry

**DOI:** 10.3390/s21217419

**Published:** 2021-11-08

**Authors:** Jing-he Wang, Jafar Tavoosi, Ardashir Mohammadzadeh, Saleh Mobayen, Jihad H. Asad, Wudhichai Assawinchaichote, Mai The Vu, Paweł Skruch

**Affiliations:** 1 School of Economics and Finance, Huaqiao University, Quanzhou 362021, China; look87@126.com; 2 Department of Electrical Engineering, Ilam University, Ilam 69315516, Iran; j.tavoosi@ilam.ac.ir; 3 Electrical Engineering Department, University of Bonab, Bonab 5551395133, Iran; a.mzadeh@ubonab.ac.ir; 4 Future Technology Research Center, National Yunlin University of Science and Technology, Douliu 64002, Taiwan; 5 Department of Physics, Faculty of Applied Sciences, Palestine Technical University, Tulkarm P.O. Box 7, Palestine; j.asad@ptuk.edu.ps; 6 Department of Electronic and Telecommunication Engineering, Faculty of Engineering, King Mongkut’s University of Technology Thonburi, Bangkok 10140, Thailand; 7 School of Intelligent Mechatronics Engineering, Sejong University, Seoul 05006, Korea; maithevu90@sejong.ac.kr; 8 Department of Automatic Control and Robotics, AGH University of Science and Technology, 30-059 Kraków, Poland; skruch@agh.edu.pl

**Keywords:** learning algorithm, fault detection, type-3 fuzzy logic, non-Gaussian noise, correntropy Kalman filter

## Abstract

The main contribution of this paper is to develop a new flowmeter fault detection approach based on optimized non-singleton type-3 (NT3) fuzzy logic systems (FLSs). The introduced method is implemented on an experimental gas industry plant. The system is modeled by NT3FLSs, and the faults are detected by comparison of measured end estimated signals. In this scheme, the detecting performance depends on the estimation and modeling performance. The suggested NT3FLS is used because of the existence of a high level of measurement errors and uncertainties in this problem. The designed NT3FLS with uncertain footprint-of-uncertainty (FOU), fuzzy secondary memberships and adaptive non-singleton fuzzification results in a powerful tool for modeling signals immersed in noise and error. The level of non-singleton fuzzification and membership parameters are tuned by maximum correntropy (MC) unscented Kalman filter (KF), and the rule parameters are learned by correntropy KF (CKF) with fuzzy kernel size. The suggested learning algorithms can handle the non-Gaussian noises that are common in industrial applications. The various types of flowmeters are investigated, and the effect of common faults are examined. It is shown that the suggested approach can detect the various faults with good accuracy in comparison with conventional approaches.

## 1. Introduction

Instrument error is defined as the error between the actual and measured values. The instant error detection in large oil and gas industries, petrochemicals, power plants, etc., has long been important, and therefore, more research has focused on these industries. At an oil refinery, for example, everything happens relatively large: liquids flow in tons per hour, temperatures are measured in the hundreds to thousands of degrees Celsius, and electricity is generated in megawatts. Thousands and millions of dollars are spent at any given time because a small or faulty system defect can cause heavy damage to the whole system and workers or major losses. Therefore, to achieve acceptable safety, remarkable durability and well efficiency sensor management should be taken to account. Due to the importance of processes, in the field of oil and gas jobs, thousands of sensors have already been installed in and around their physical systems.

Another source of error is the data logger system. In most data acquisition systems (DASs), there exist both systematic (e.g., nonlinearity) and random (e.g., electronic noise) error effects simultaneously. While systematic errors are a relatively stable feature of a DAS, random errors may be smaller or larger in different situations, and it is important to understand how they reduce the overall system performance. It is obvious that the higher number of sampling times and the number of bits used in the sampling process lead to the higher accuracy of analog-to-digital conversion signal. The difference between the original signal and the digitized signal is called the quantization error. Furthermore, the difference between the highest and lowest levels of signals that can be converted to digital by a certain number of bits is called Dynamic Range. Quantization error distorts the digital signal. This is because the analog signal at the sampling points must be rounded to the nearest value that can be detected and converted in the system. The higher number of bits used in the conversion process causes the sampled values to be closer to the original values, and therefore, the difference between the digital signal and the original analog signal becomes smaller [1].

## 2. Literature Review

The sensor fault detection (FD) is an important issue in various studies. Many researchers have paid attention to this problem. For example, in [2], some main features are identified by FLSs to determine the healthy conditions, and based on the extracted features, a fault detection method is proposed. In [3], by the curve fitting approach, the phase difference is detected in Coriolis flowmeters, and its accuracy is compared with Fourier-based methods. The variable selection method by the use of the causality approach is studied in [4] for the FD problem in the gas industry. The results are compared with filter-based and embedded-based methods, and it is shown that model identification by the suggested variable selection approach improves the FD performance. In [5], the fault of pressure sensors is studied, and a dynamic model is optimized to predict the pressure. In this paper, a pressure observe is designed to predict the sensor output, and the convergence is theoretically analyzed. In [6], by the use of Kalman filter, the flow is estimated, and by the Lyapunov approach, the stability is investigated. In [7], a wireless FD scheme is developed to detect the current fault in motors, and its energy consumption and detection accuracy are investigated. In [8], the detectability problem is taken into account, and the sensor locations are optimized to improve the FD accuracy. The FD of virtual sensors is studied in [9], and the accuracy of linear regression approaches is investigated. Considering various sensors, such as temperature and airflow virtual sensors, the suggested method is examined on a real building in Denmark, and it is shown that abnormal oscillation in temperature is strongly detected. In [10], a fiber-optic approach is introduced, and its accuracy and maintenance cost is studied. The variance shift scheme is formulated in [11] to construct an FD method for a navigation system. In [12], an FD system is developed for switching systems with uncertain switching modes, and the accuracy of the FD method is examined on a three-tank system. A multimodal FD approach is designed in [13] to detect shunt failures. The various FD methods for heating/cooling systems are reviewed in [14]. In [15], by the use of historical data, the outputs of sensors are predicted, and by comparison with the measured data, the fault is detected. The frequency estimation approach is developed in [16] for an FD problem.

Recently, the machine learning methods and artificial intelligence techniques have been developed for various applications, such as helicopter hydraulic FD problems, medical diagnosis systems, risk assessment applications, and so on [17,18,19,20]. These approaches have also been employed for FD problems in a few studies. For example, in [21], a neural network (NN) is used for modeling the signal dynamics and prediction, and based on the NN model, an FD scheme is developed for pneumatic valves. In [22], the genetic algorithm is suggested for online optimization of an NN and designing an FD system. In [23], an NN-based FD is proposed for hydraulic systems, and it is shown that using NNs improves the accuracy. A deep learned NN scheme is designed in [24] for the FD problem in gas turbines. In [25], an FLS is used to approximate the flow pattern, and then based on the FLS model, the flow is predicted, and an FD scheme is designed. In [26], a recurrent NN is optimized to detect the fault in the drilling process of gas industries. An FD system based on NNs is suggested in [27] for reactive distillation, and its diagnosis capability is compared with conventional approaches. The support vector machine is developed in [28] for modeling flow patterns and fault detection. In [29], a simple NN is learned by the backpropagation approach, and it is used for the prediction of fluidization velocity and FD in gas industries.

The type-3 FLSs (T3-FLSs) have been recently developed to improve the estimation capability of conventional FLSs and improve the robustness against noisy environments. T3-FLSs have been employed in various practical problems, such as energy management [30], gyroscopes [31], forecasting problems [32], industrial 5G converters [33], and so on. In the aforementioned studies, it is verified that the developed T3-FLSs give a better performance in noisy conditions. However, it has not been used for fault detection problems in industrial systems.

In most of the above reviewed FD methods, the data uncertainties are neglected, and the signal dynamic/pattern is estimated by the noisy measured data. Furthermore, the simple learning methods have been used, and they cannot handle the non-Gaussian noise. The non-Gaussian noise is very prevalent in industrial applications. In this paper, by the use of a novel approach based on the non-singleton fuzzification, the uncertainty of historical measurement data is also considered. A type-3 FLS with high estimation capability in a noisy environment is presented to predict the signal pattern and fault detection well. The nonlinear Kalman approach with a fuzzy kernel size is formulated for learning NT3FLS in the presence of non-Gaussian measurement noise.

## 3. General View on Suggested Approach

**Figure 1:**
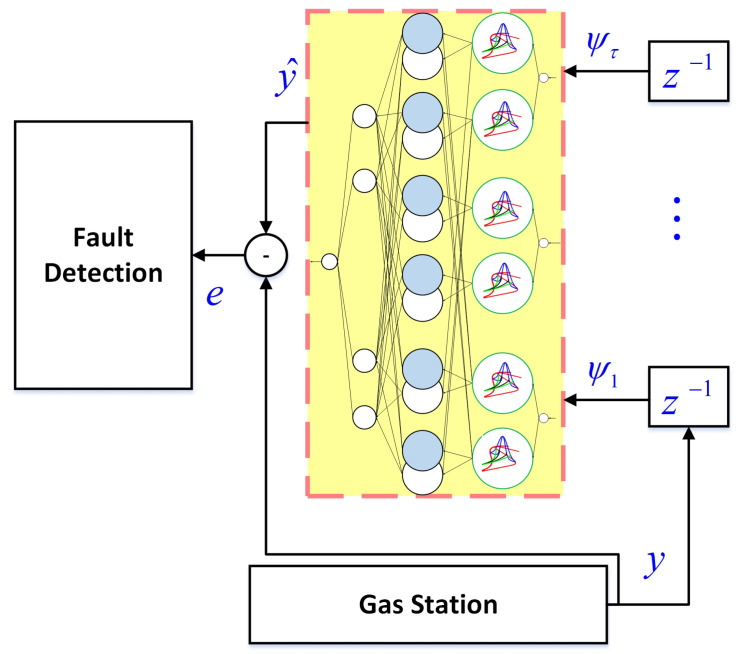
General scheme of the type-3 FLS fault detection algorithm.

The general view of the suggested approach is shown in Figure 1. The main signals are modeled by the designed NT3-FLS, and then by comparing the measurement signals and approximated signals, the faults are detected.

## 4. Non-Singleton Interval Type-3 FLS

**Figure 2:**
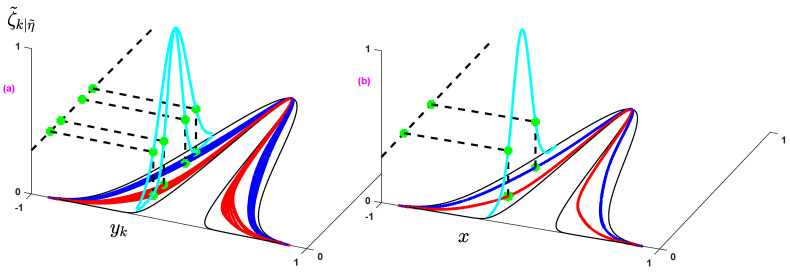
Comparison of fuzzy sets: (**a**) type-3 fuzzy set, (**b**): type-2 fuzzy set.

**Figure 3:**
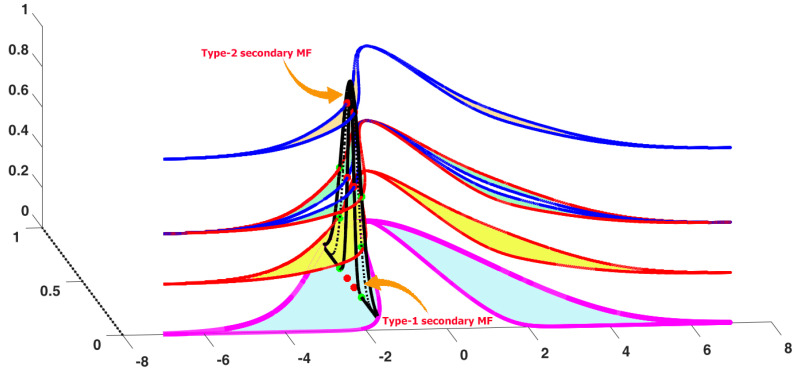
Horizontal slice for a type-3 MF.

The type-3 FLSs can model a higher level of uncertainties in contrast to the type-2 counterparts because the secondary membership and also the upper and lower uncertainties in a type-3 fuzzy set are not crisp values, but they are fuzzy sets. To better see the difference, consider a horizontal slice in type-3 and general type-2 fuzzy sets, as shown in Figure 2, we see that the bounds of uncertainties (upper and lower of primary memberships) in the type-3 fuzzy set are not crisp values, but they also interval fuzzy sets. Furthermore, in comparison with general type-2 MFs, the type-3 MFs have more degrees of freedom. As shown in Figure 3, a horizontal slice in the type-3 MFs can be represented by two slices in the general type-2 counterparts. In other words, we theoretically see that the type-3 MFs can model more levels of uncertainties because of their uncertain secondary memberships and uncertain FOU, and also from a functionality view point, we see that the type-3 MFs have more degrees of freedom. The basic idea for type-3 FLSs is presented in [34]. In this paper, the non-singleton form of basic type-3 FLSs is formulated in a fault detection scheme.

**Remark** **1.** *It should be noted that the various horizontal slices are used in the computation of the output of FLS, then the effects of variation of the footprint of uncertainty (FOU) are considered in computations.*

The dynamics of the case-study plant are completely unknown. Furthermore, the measured data set is stained with noise. Then in this problem, we are facing a high level of uncertainty, and the type-3 FLSs with better uncertainty modeling capability is used. Furthermore, the non-singleton fuzzification is used to handle the uncertainty of the measured data.

**Figure 4:**
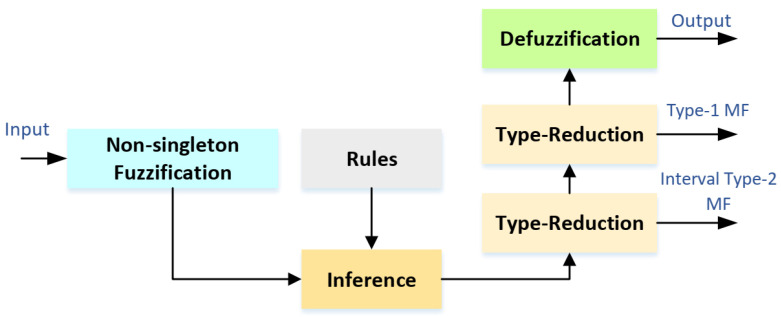
The general structure of NT3FLS.

**Figure 5:**
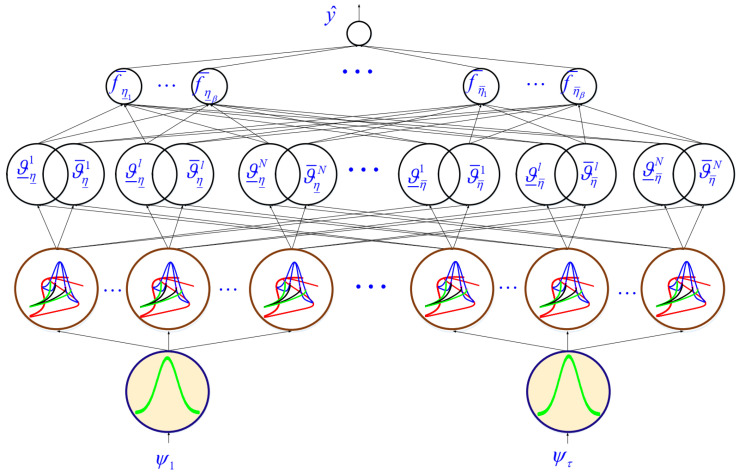
The detailed diagram of NT3FLS.

The structure of the NT3FLS for modeling the signal dynamic is described in this section. The general structure is given in Figure 4. The detailed scheme is depicted in Figure 5. The estimated signal is computed as follows.

**Figure 6:**
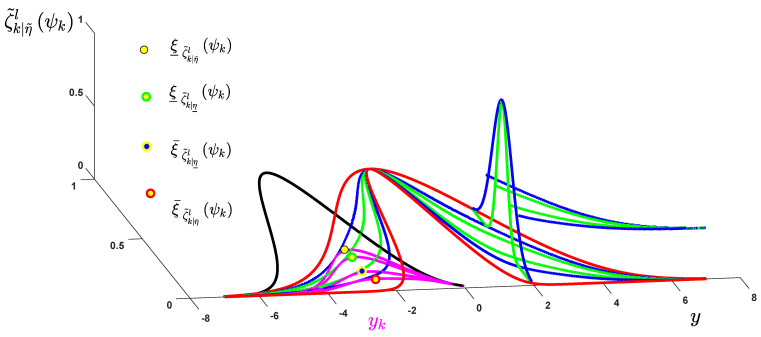
Non-singleton fuzzification.

(1) The inputs of NT3FLS at sample time *t* are the value of the target signal at sample time *t* and its previous data $\psi \left(t-1\right),\ldots ,\psi \left(t-\tau \right)$. The inputs are denoted by $\psi_{1},\ldots ,\psi_{\tau }$, where τ is the number of inputs.

(2) In the second step, the non-singleton fuzzification is computed. Gaussian membership functions (MFs) are considered to handle the input uncertainties. The standard division (SD) of this MF represents the uncertainty level of the measured signal. The Gaussian MFs are used because they are derivable, and the non-singleton fuzzification in Gaussian MFs is also much easier in contrast to triangular counterparts.

(3) The memberships for inputs $\psi_{1},\ldots ,\psi_{\tau }$ are computed. The memberships of ${\tilde{\zeta }}_{k}^{l}$ (l−th MF for k−th input) at the slice level $\underset{\bar{}}{\eta },\bar{\eta }$ (see Figure 6) are computed as:

(1) $${\bar{\xi }}_{{\tilde{\zeta }}_{k|\bar{\eta }}^{l}}\left(\psi_{k}\right)=\exp\left(-\frac{{\left({\bar{\psi }}_{k|\bar{\eta }}^{l}-c_{{\tilde{\zeta }}_{k}^{l}}\right)}^{2}}{{\bar{\sigma }}_{{\tilde{\zeta }}_{k|\bar{\eta }}^{l}}^{2}}\right)$$

(2) $${\bar{\xi }}_{{\tilde{\zeta }}_{k|\underset{\bar{}}{\eta }}^{l}}\left(\psi_{k}\right)=\exp\left(-\frac{{\left({\bar{\psi }}_{k|\underset{\bar{}}{\eta }}^{l}-c_{{\tilde{\zeta }}_{k}^{l}}\right)}^{2}}{{\bar{\sigma }}_{{\tilde{\zeta }}_{k|\underset{\bar{}}{\eta }}^{l}}^{2}}\right)$$

(3) $${\underset{\bar{}}{\xi }}_{{\tilde{\zeta }}_{k|\bar{\eta }}^{l}}\left(\psi_{k}\right)=\exp\left(-\frac{{\left({\underset{\bar{}}{\psi }}_{k|\bar{\eta }}^{l}-c_{{\tilde{\zeta }}_{k}^{l}}\right)}^{2}}{{\underset{\bar{}}{\sigma }}_{{\tilde{\zeta }}_{k|\bar{\eta }}^{l}}^{2}}\right)$$

(4) $${\underset{\bar{}}{\xi }}_{{\tilde{\zeta }}_{k|\underset{\bar{}}{\eta }}^{l}}\left(\psi_{k}\right)=\exp\left(-\frac{{\left({\underset{\bar{}}{\psi }}_{k|\underset{\bar{}}{\eta }}^{l}-c_{{\tilde{\zeta }}_{k}^{l}}\right)}^{2}}{{\underset{\bar{}}{\sigma }}_{{\tilde{\zeta }}_{k|\underset{\bar{}}{\eta }}^{l}}^{2}}\right)$$

where $c_{{\tilde{\zeta }}_{k}^{l}}$ denotes the center of ${\tilde{\zeta }}_{k}^{l}$, ${\bar{\sigma }}_{{\tilde{\zeta }}_{k|\bar{\eta }}^{l}}$/${\underset{\bar{}}{\sigma }}_{{\tilde{\zeta }}_{k|\bar{\eta }}^{l}}^{2}$ represent the upper/lower SDs for slice level $\bar{\eta }$, and ${\bar{\sigma }}_{{\tilde{\zeta }}_{k|\underset{\bar{}}{\eta }}^{l}}$ and ${\underset{\bar{}}{\sigma }}_{{\tilde{\zeta }}_{k|\underset{\bar{}}{\eta }}^{l}}$ are the SD at slice level $\underset{\bar{}}{\eta }$. The fuzzification terms ${\bar{\psi }}_{k|\bar{\eta }}^{l}$, ${\bar{\psi }}_{k|\underset{\bar{}}{\eta }}^{l}$, ${\underset{\bar{}}{\psi }}_{k|\bar{\eta }}^{l}$ and ${\underset{\bar{}}{\psi }}_{k|\underset{\bar{}}{\eta }}^{l}$ are obtained as:

(5) $${\bar{\psi }}_{k|\bar{\eta }}^{l}=\frac{{\bar{\sigma }}_{{\tilde{\zeta }}_{k|\bar{\eta }}^{l}}^{2}\psi_{k}+\sigma_{\psi }^{2}c_{{\tilde{\zeta }}_{k}^{l}}}{{\bar{\sigma }}_{{\tilde{\zeta }}_{k|\bar{\eta }}^{l}}^{2}+\sigma_{\psi }^{2}}$$

(6) $${\bar{\psi }}_{k|\underset{\bar{}}{\eta }}^{l}=\frac{{\bar{\sigma }}_{{\tilde{\zeta }}_{k|\underset{\bar{}}{\eta }}^{l}}^{2}\psi_{k}+\sigma_{\psi }^{2}c_{{\tilde{\zeta }}_{k}^{l}}}{{\bar{\sigma }}_{{\tilde{\zeta }}_{k|\underset{\bar{}}{\eta }}^{l}}^{2}+\sigma_{\psi }^{2}}$$

(7) $${\underset{\bar{}}{\psi }}_{k|\bar{\eta }}^{l}=\frac{{\underset{\bar{}}{\sigma }}_{{\tilde{\zeta }}_{k|\bar{\eta }}^{l}}^{2}\psi_{k}+\sigma_{\psi }^{2}c_{{\tilde{\zeta }}_{k}^{l}}}{{\underset{\bar{}}{\sigma }}_{{\tilde{\zeta }}_{k|\bar{\eta }}^{l}}^{2}+\sigma_{\psi }^{2}}$$

(8) $${\underset{\bar{}}{\psi }}_{k|\underset{\bar{}}{\eta }}^{l}=\frac{{\underset{\bar{}}{\sigma }}_{{\tilde{\zeta }}_{k|\underset{\bar{}}{\eta }}^{l}}^{2}\psi_{k}+\sigma_{\psi }^{2}c_{{\tilde{\zeta }}_{k}^{l}}}{{\underset{\bar{}}{\sigma }}_{{\tilde{\zeta }}_{k|\underset{\bar{}}{\eta }}^{l}}^{2}+\sigma_{\psi }^{2}}$$

(4) The l−th rule is written as:

(9) $$\begin{array}{c} \mathrm{If}\;\psi_{1}\;\;\mathrm{is}\;\;{\tilde{\zeta }}_{1}^{l}\;\;\mathrm{and}\;\ldots \;\;\psi_{\tau }\;\;\mathrm{is}\;\;{\tilde{\zeta }}_{\tau }^{l}\;\;\mathrm{Then}\;\;\\ \;\;\;\;\;\;\;\;\;\;\;\;\;\;\;\;\;\;\;\;\;\;\;\;\;\;\;\;\;\;\;\;\;\;\;\;\;\;\;\;\;\;\;\;\;{\hat{y}}_{l}\in \left[{\underset{\bar{}}{w}}_{l},{\bar{w}}_{l}\right] \end{array}$$

The rule firings $\bar{\vartheta }$ and $\underset{\bar{}}{\vartheta }$ at slice levels $\underset{\bar{}}{\eta }$ and $\bar{\eta }$ are computed as:

(10) $${\bar{\vartheta }}_{\bar{\eta }}^{l}={\bar{\xi }}_{{\tilde{\zeta }}_{1|\bar{\eta }}^{l}}\left(\psi_{1}\right)\times \cdots \times {\bar{\xi }}_{{\tilde{\zeta }}_{\tau |\bar{\eta }}^{l}}\left(\psi_{\tau }\right)$$

(11) $${\bar{\vartheta }}_{\underset{\bar{}}{\eta }}^{l}={\bar{\xi }}_{{\tilde{\zeta }}_{1|\underset{\bar{}}{\eta }}^{l}}\left(\psi_{1}\right)\times \cdots \times {\bar{\xi }}_{{\tilde{\zeta }}_{\tau |\underset{\bar{}}{\eta }}^{l}}\left(\psi_{\tau }\right)$$

(12) $${\underset{\bar{}}{\vartheta }}_{\bar{\eta }}^{l}={\underset{\bar{}}{\xi }}_{{\tilde{\zeta }}_{1|\bar{\eta }}^{l}}\left(\psi_{1}\right)\times \cdots \times {\underset{\bar{}}{\xi }}_{{\tilde{\zeta }}_{\tau |\bar{\eta }}^{l}}\left(\psi_{\tau }\right)$$

(13) $${\underset{\bar{}}{\vartheta }}_{\underset{\bar{}}{\eta }}^{l}={\underset{\bar{}}{\xi }}_{{\tilde{\zeta }}_{1|\underset{\bar{}}{\eta }}^{l}}\left(\psi_{1}\right)\times \cdots \times {\underset{\bar{}}{\xi }}_{{\tilde{\zeta }}_{\tau |\underset{\bar{}}{\eta }}^{l}}\left(\psi_{\tau }\right)$$

(5) By type-reduction of [34], $\hat{g}\left(\psi |\theta \right)$ is:

(14) $$\hat{y}\left(\psi |\theta \right)=\theta^{T}\lambda$$

where $\theta ={\left[{\underset{\bar{}}{w}}_{1},\ldots ,{\underset{\bar{}}{w}}_{N},{\bar{w}}_{1},\ldots ,{\bar{w}}_{N}\right]}^{T}$ denotes the vector of consequent parameters and $\lambda ={\left[{\underset{\bar{}}{\lambda }}_{1},\ldots ,{\underset{\bar{}}{\lambda }}_{N},{\bar{\lambda }}_{1},\ldots ,{\bar{\lambda }}_{N}\right]}^{T}$ and ${\bar{\lambda }}_{l}$ and ${\underset{\bar{}}{\lambda }}_{l}$ are as:

(15) $${\bar{\lambda }}_{l}=\frac{\sum_{N=1}^{\beta }{\underset{\bar{}}{\eta }}_{h}{\bar{f}}_{{\underset{\bar{}}{\eta }}_{h}}^{l}}{\sum_{h=1}^{\beta }\left({\bar{\eta }}_{h}+{\underset{\bar{}}{\eta }}_{h}\right)}+\frac{\sum_{h=1}^{\beta }{\bar{\eta }}_{h}{\bar{f}}_{{\bar{\eta }}_{h}}^{l}}{\sum_{h=1}^{\beta }\left({\bar{\eta }}_{h}+{\underset{\bar{}}{\eta }}_{h}\right)}$$

(16) $${\underset{\bar{}}{\lambda }}_{l}=\frac{\sum_{N=1}^{\beta }{\underset{\bar{}}{\eta }}_{h}{\underset{\bar{}}{f}}_{{\underset{\bar{}}{\eta }}_{h}}^{l}}{\sum_{h=1}^{\beta }\left({\bar{\eta }}_{h}+{\underset{\bar{}}{\eta }}_{h}\right)}+\frac{\sum_{h=1}^{\beta }{\bar{\eta }}_{h}{\underset{\bar{}}{f}}_{{\bar{\eta }}_{h}}^{l}}{\sum_{h=1}^{\beta }\left({\bar{\eta }}_{h}+{\underset{\bar{}}{\eta }}_{h}\right)}$$

where, ${\bar{f}}_{{\underset{\bar{}}{\eta }}_{h}}^{l}$, ${\bar{f}}_{{\bar{\eta }}_{h}}^{l}$, ${\underset{\bar{}}{f}}_{{\underset{\bar{}}{\eta }}_{h}}^{l}$ and ${\underset{\bar{}}{f}}_{{\bar{\eta }}_{h}}^{l}$ are as:

(17) $${\bar{f}}_{{\underset{\bar{}}{\eta }}_{h}}^{l}=\frac{{\bar{\vartheta }}_{{\underset{\bar{}}{\eta }}_{h}}^{l}}{\sum_{l=1}^{N}\left({\bar{\vartheta }}_{{\underset{\bar{}}{\eta }}_{h}}^{l}+{\underset{\bar{}}{\vartheta }}_{{\underset{\bar{}}{\eta }}_{h}}^{l}\right)}$$

(18) $${\bar{f}}_{{\bar{\eta }}_{h}}^{l}=\frac{{\bar{\vartheta }}_{{\bar{\eta }}_{h}}^{l}}{\sum_{l=1}^{N}\left({\bar{\vartheta }}_{{\bar{\eta }}_{h}}^{l}+{\underset{\bar{}}{\vartheta }}_{{\bar{\eta }}_{h}}^{l}\right)}$$

(19) $${\underset{\bar{}}{f}}_{{\underset{\bar{}}{\eta }}_{h}}^{l}=\frac{{\underset{\bar{}}{\vartheta }}_{{\underset{\bar{}}{\eta }}_{h}}^{l}}{\sum_{l=1}^{N}\left({\bar{\vartheta }}_{{\underset{\bar{}}{\eta }}_{h}}^{l}+{\underset{\bar{}}{\vartheta }}_{{\underset{\bar{}}{\eta }}_{h}}^{l}\right)}$$

(20) $${\underset{\bar{}}{f}}_{{\bar{\eta }}_{h}}^{l}=\frac{{\underset{\bar{}}{\vartheta }}_{{\bar{\eta }}_{h}}^{l}}{\sum_{l=1}^{N}\left({\bar{\vartheta }}_{{\bar{\eta }}_{h}}^{l}+{\underset{\bar{}}{\vartheta }}_{{\bar{\eta }}_{h}}^{l}\right)}$$

## 5. Learning Algorithm

In this section, the rules and MF parameters and fuzzification level are optimized.

### 5.1. Rule Parameters

To adjust the consequent (rule) parameters of NT3FLS, the CKF is applied as:

(21) $$\theta_{c}\left(t\right)=\theta_{c}\left(t-1\right)+\gamma \left(t\right)\left(y-\lambda^{T}\left(t-1\right)\theta_{c}\left(t-1\right)\right)$$

where

(22) $$\gamma \left(t\right)={\left(I_{M}+\lambda^{T}\lambda \left(t\right)\lambda \right)}^{-1}\lambda \left(t\right)\lambda$$

(23) $$\lambda \left(t\right)=F_{\hat{\sigma }}\left(∥y-\lambda^{T}\left(t-1\right)\theta_{c}\left(t-1\right)∥\right)$$

where $F_{\hat{\sigma }}$ denotes the kernel function. The kernel size σ is obtained by an FLS [35].

### 5.2. Antecedent Parameters

The centers of MFs $c_{\tilde{\zeta }}$ and the level of fuzzifications $\sigma_{\psi }$ are tuned by MUKF as follows.

(1) The designed NT3FLS is rewritten as:
  (24) $$\begin{array}{c} \theta_{a}\left(t\right)=\theta_{a}\left(t-1\right)+\Phi \left(t\right)\\ \hat{y}\left(t\right)=\mathrm{NT}3\mathrm{FLS}\left(\psi \left(t\right)\left|\theta_{a}\left(t\right)\right|\theta_{c}\left(t\right)\right)+ℜ\left(t\right) \end{array}$$
  where,
  (25) $$\psi =\left[\psi_{1},\ldots ,\psi_{\tau }\right]$$
  (26) $$\theta_{a}={\left[c_{{\tilde{\zeta }}_{1}^{1}},\ldots ,c_{{\tilde{\zeta }}_{1}^{N}},\ldots ,c_{{\tilde{\zeta }}_{n}^{1}},\ldots ,c_{{\tilde{\zeta }}_{n}^{N}},\sigma_{\psi }\right]}^{T}$$
  where, $\theta_{c}\left(t\right)$ is vector of rule (consequent) parameters, $c_{{\tilde{\zeta }}_{i}^{j}}$ is the center of j−th MF for i−th input $\psi_{i}$, $\sigma_{\psi }$ is the level of fuzzification and *N* is the number of MFs.
(2) Initialize the vector $\theta_{a}$ and $\theta_{c}$.
(3) The sigma-points are defined as:
  (27) $$\begin{array}{c} {\tilde{\theta }}_{a}^{\kappa }\left(t\right)=\theta_{a}\left(t\right)\\ \;\;\;\;\;\;\;\;\;\;\;\;\;+{\left(\sqrt{\left(d_{a}+\lambda \right)\varphi \left(t\right)}\right)}^{\kappa },\;\kappa =1,\ldots ,d_{a}\\ {\tilde{\theta }}_{a}^{\kappa }\left(t\right)=\theta_{a}\left(t\right)\\ \;\;\;\;\;\;\;\;\;\;\;\;\;-{\left(\sqrt{\left(d_{a}+\lambda \right)\varphi \left(t\right)}\right)}^{\kappa -d_{a}},\;\kappa =d_{a}+1,\ldots ,2d_{a}\\ {\tilde{\theta }}_{a}^{\kappa }\left(t\right)=\theta_{a}\left(t\right),\;\;\kappa =0 \end{array}$$
  where $d_{a}$ denotes the number of elements of vector $\theta_{a}$, φ is covariance matrix of $\theta_{a}$, ${\left(\sqrt{\left(d_{a}+\lambda \right)\varphi \left(t\right)}\right)}^{\kappa }$ is the κ-th column of ${\left(\sqrt{\left(d_{a}+\lambda \right)\varphi \left(t\right)}\right)}^{\kappa }$ and λ denotes the turning factor.
(4) Compute $\hat{y}\left(t\right)$ (estimated signal) as:
  (28) $$\begin{array}{c} \hat{y}\left(t\right)=\frac{1}{2\left(d_{a}+\lambda \right)}\sum_{\kappa =1}^{2d_{a}}\mathrm{NT}3\mathrm{FLS}\left(\psi \left(t\right)\left|{\tilde{\theta }}_{a}^{\kappa }\left(t\right)\right|\theta_{c}\left(t\right)\right)+\\ \;\;\;\frac{\lambda }{d_{a}+\lambda }\mathrm{NT}3\mathrm{FLS}\left(\psi \left(t\right)\left|{\tilde{\theta }}_{a}^{0}\left(t\right)\right|\theta_{c}\left(t\right)\right) \end{array}$$
(5) Compute $\tilde{\varphi }$ and $\tilde{R}$ as:
  (29) $$\begin{array}{c} {\tilde{\lambda }}_{a}=\Upsilon_{\Lambda }\psi_{\psi }^{-1}\Upsilon_{\Lambda }^{T}\\ \tilde{R}=\Upsilon_{r}\psi_{y}^{-1}\Upsilon_{r}^{T} \end{array}$$
  where
  (30) $$\left[\begin{array}{cc} \varphi & 0\\ 0 & R \end{array}\right]=\left[\begin{array}{cc} \Upsilon_{\Lambda }\Upsilon_{\Lambda }^{T} & 0\\ 0 & \Upsilon_{r}\Upsilon_{r}^{T} \end{array}\right]=\Upsilon \Upsilon^{T}$$
  (31) $$\begin{array}{c} \psi_{\psi }^{}=diag\left(G_{\sigma }\left(\chi_{1}\right),\ldots ,G_{\sigma }\left(\chi_{d_{a}}\right)\right)\\ \psi_{y}^{}=diag\left(G_{\sigma }\left(\chi_{d_{a}+1}\right),\ldots ,G_{\sigma }\left(\chi_{L}\right)\right) \end{array}$$
  (32) $$\chi =\Upsilon^{-1}\left[\begin{array}{c} \theta_{a}\\ y-\hat{y}+H\theta_{a} \end{array}\right]-\Upsilon^{-1}\left[\begin{array}{c} I\\ H \end{array}\right]\theta_{a}$$
  (33) $$H=\lambda_{xy}^{T}\lambda^{-1}$$
  (34) $$\begin{array}{c} \lambda_{xy}^{T}=\frac{1}{2\left(d_{a}+\lambda \right)}\sum_{\kappa =1}^{2d_{a}}{{\tilde{\theta }}_{a}^{\kappa }\left[\mathrm{NT}3\mathrm{FLS}\left(\psi |{\tilde{\theta }}_{a}^{\kappa }|\theta_{c}\right)\right]}^{T}-\theta_{a}^{}\hat{y}\\ \;\;\;\frac{\lambda }{d_{a}+\lambda }{\tilde{\theta }}_{a}^{0}{\left[\mathrm{NT}3\mathrm{FLS}\left(\psi |{\tilde{\theta }}_{a}^{0}|\theta_{c}\right)\right]}^{T} \end{array}$$
  *L* represents the dimension of χ, and $G_{\sigma }\left(\chi \right)$ is:
  (35) $$G_{\sigma }\left(\chi \right)\;=\exp\left(-\frac{\chi^{2}}{2\sigma }\right)$$
  The value of σ is obtained by a FLS [35].
(6) $\theta_{a}$ and φ are updated as:
  (36) $$\theta_{a}\left(t+1\right)=\theta_{a}\left(t\right)+\gamma \left(t\right)\left(y\left(t\right)-\hat{y}\left(t\right)\right)$$
  (37) $$\begin{array}{c} \varphi \left(t+1\right)=\left(I-\gamma \left(t\right)H\left(t\right)\right)\varphi \left(t\right){\left(I-\gamma \left(t\right)H\left(t\right)\right)}^{T}\\ \;\;\;\;\;\;\;\;\;\;\;\;\;\;\;\;\;\;\;\;\;\;\;\;+\gamma \left(t\right)R\left(t\right)\gamma^{T}\left(t\right) \end{array}$$
  where $\gamma \left(t\right)$ is
  (38) $$\gamma \left(t\right)={\tilde{\lambda }}_{a}\left(t\right)H^{T}\left(t\right){\left(\tilde{R}\left(t\right)+H\left(t\right){\tilde{\lambda }}_{a}\left(t\right)H^{T}\left(t\right)\right)}^{-1}$$

## 6. Data Description

**Figure 7:**
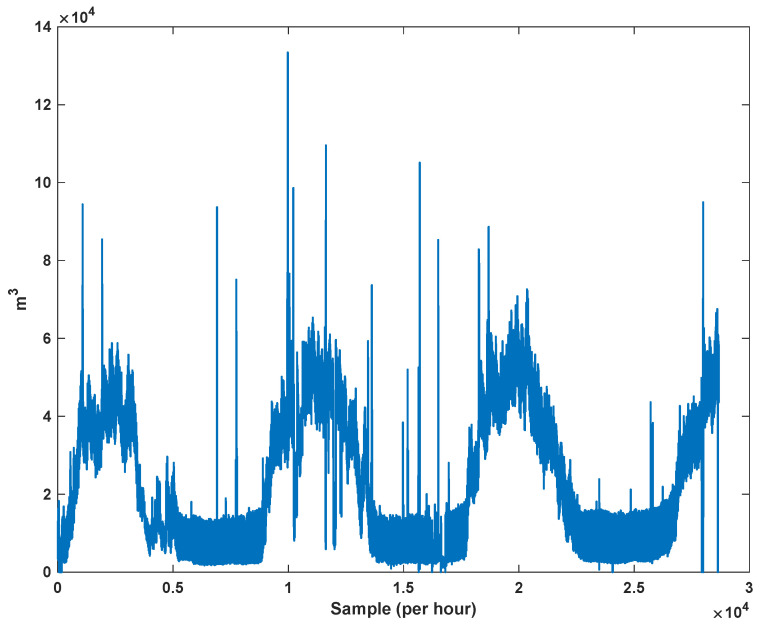
The peak of gas consumption in per hour.

The trajectory of the peak of consumption per hour is depicted in Figure 7. The total number of data is 28,673. We see that the peak of consumption is increased each year. The main reason is the changing of lifestyle and the development of gas supply for various areas.

**Figure 8:**
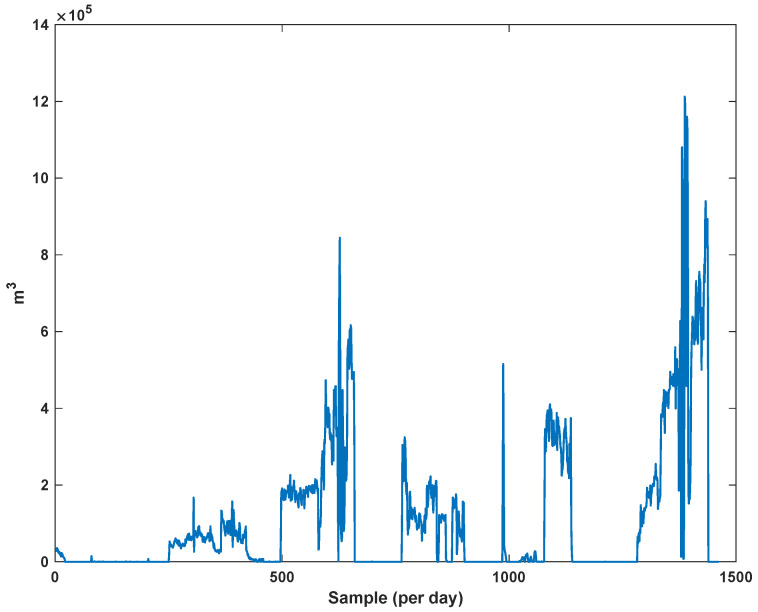
The trajectory of consumption line 1.

**Figure 9:**
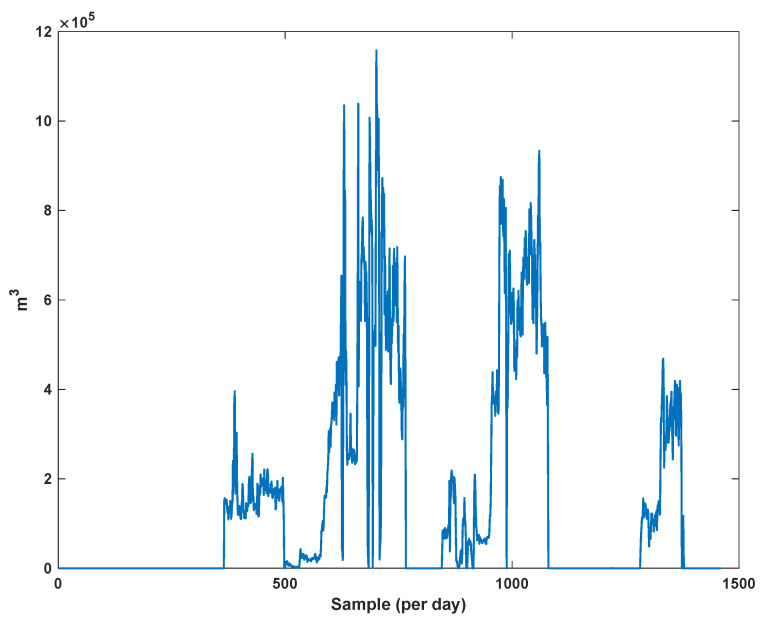
The trajectory of consumption line 2.

**Figure 10:**
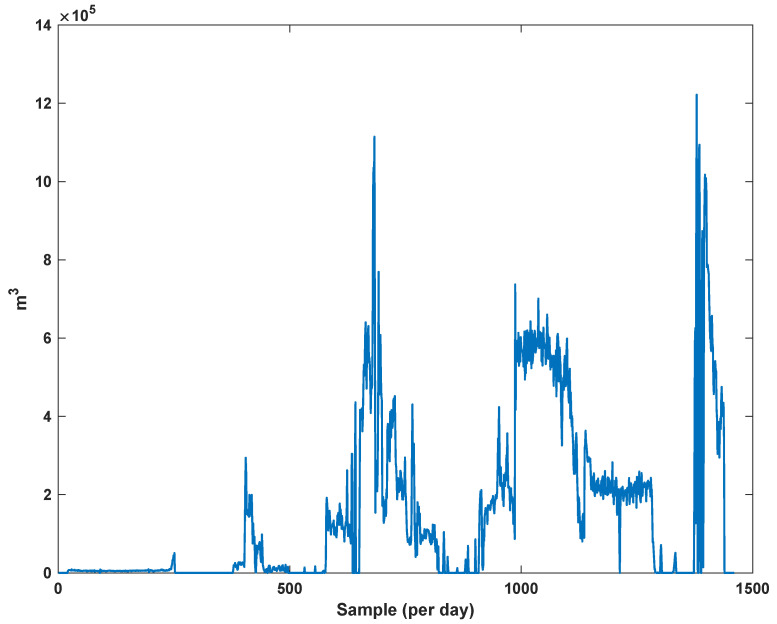
The trajectory of consumption line 3.

The diagrams of the consumption lines in the Ilam CGS station are examined below. For each line, 1460 data have been recorded. Data are related to the daily consumption from 21 March 2017 to 20 March 2021. The trajectories for consumption lines 1–3 and total consumption are shown in Figure 8, Figure 9 and Figure 10. It is seen that consumption is rapidly changed. For example, around the 700th day, the total consumption has suddenly reached 30% of its value.

## 7. Simulation

### 7.1. Modeling

**Figure 11:**
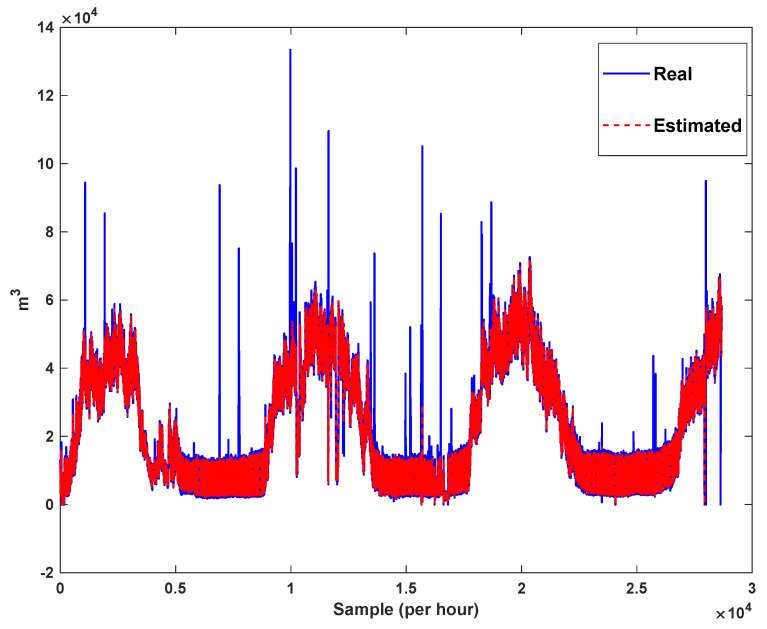
Modeling peak of consumption in per hour.

**Figure 12:**
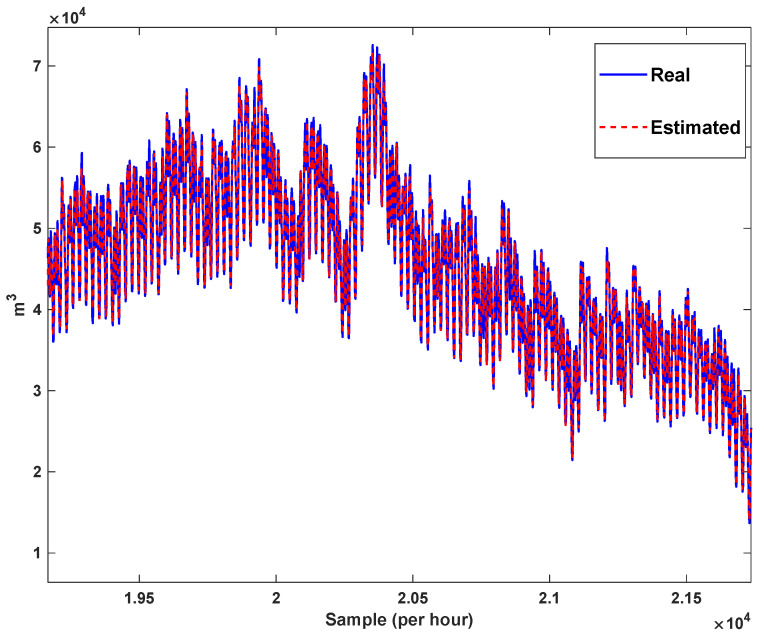
Modeling peak of consumption in per hour: Zoomed view.

**Figure 13:**
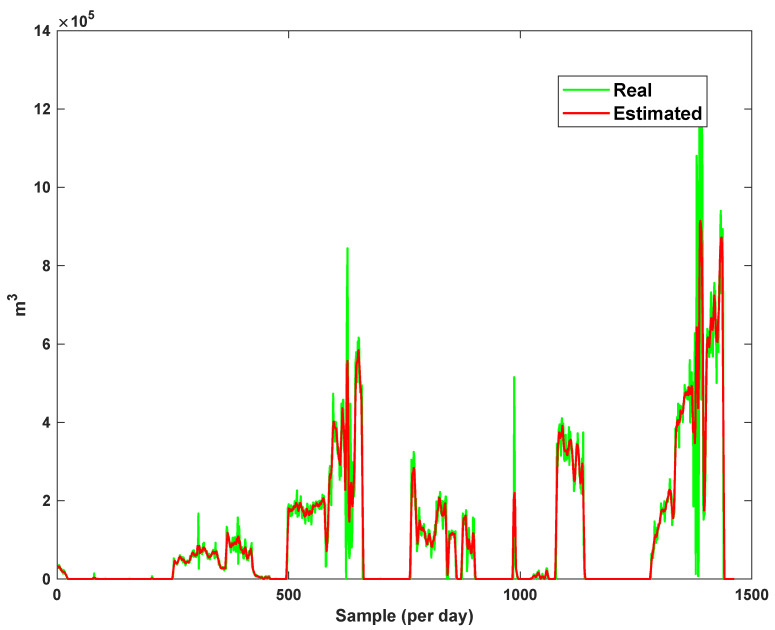
Modeling of consumption for line 1.

**Figure 14:**
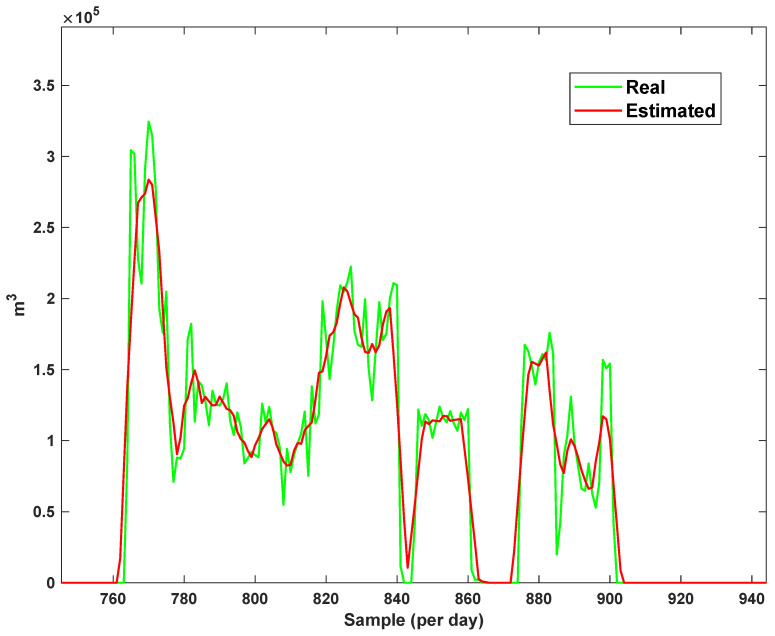
Modeling of consumption for line 1: Zoomed view.

**Figure 15:**
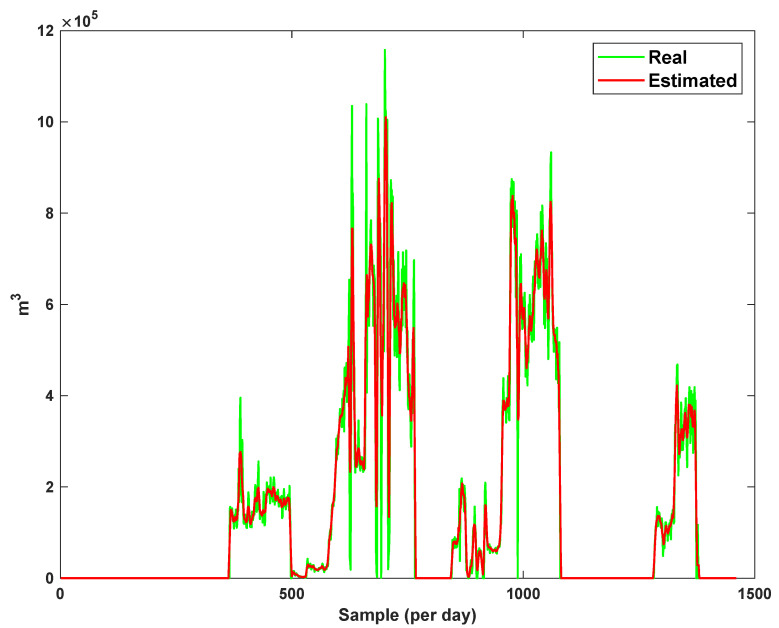
Modeling of consumption for line 2.

**Figure 16:**
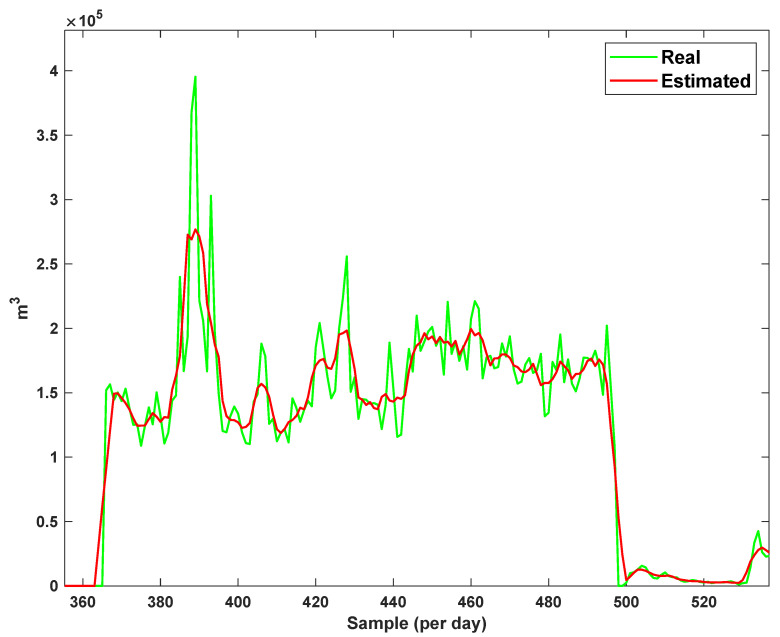
Modeling of consumption for line 2: Zoomed view.

**Figure 17:**
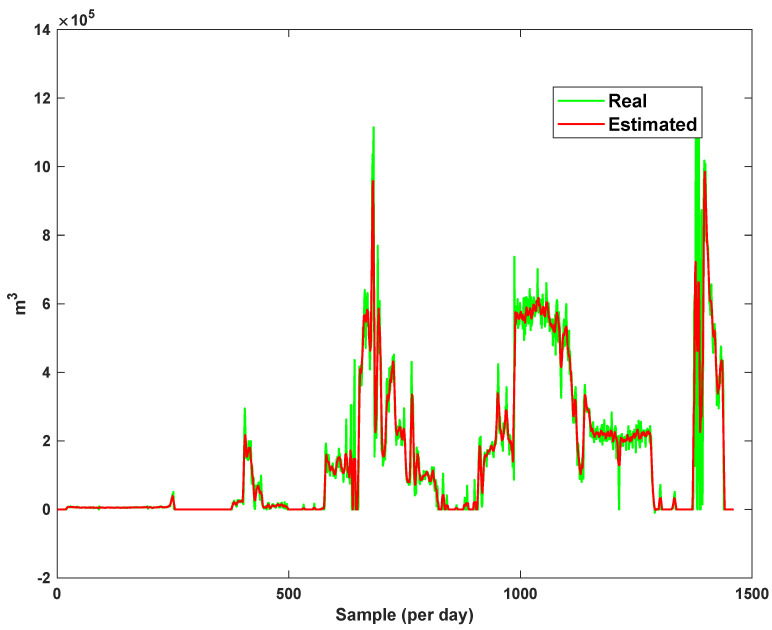
Modeling of consumption for line 3.

**Figure 18:**
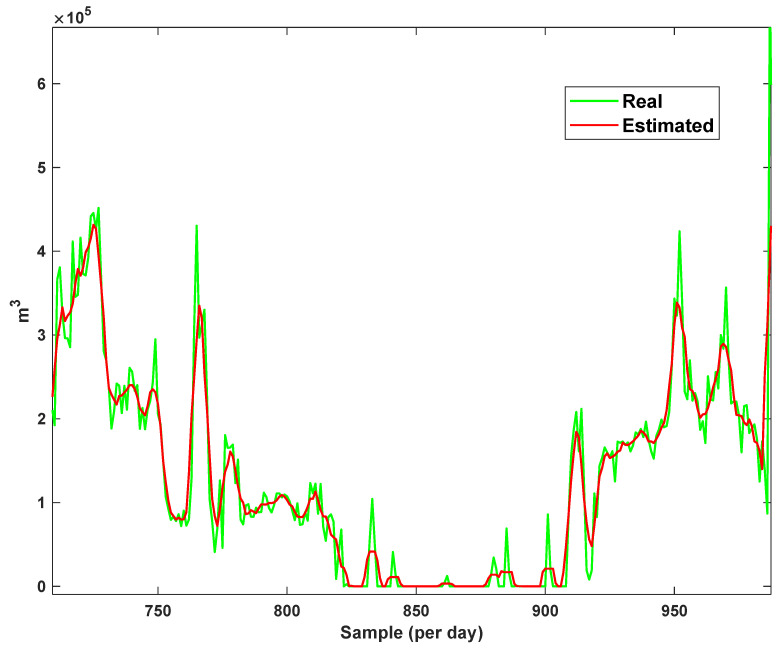
Modeling of consumption for line 3: Zoomed view.

The estimation capability of the suggested NT3FLS is examined. For each input, 11 MFs are considered. The centers of inputs are located in 0,0.1,0.2,…,1. It should be noted that the input signals of NT3FLS are normalized between 0 and 1 by dividing the input signals by their upper bounds. The trajectory of the estimated peak of consumption and the actual peak of consumption are shown in Figure 11. We observe that the estimated peak tracks the actual signal with reasonable accuracy. The zoomed view is depicted in Figure 12. The modeling performance of consumption for Line 1 and its zoomed view are depicted in Figure 13 and Figure 14. Similarly, for lines 2–3, the modeling performance zoomed views are depicted in Figure 15, Figure 16, Figure 17 and Figure 18. The simulations show that the suggested approach is effective, and the estimated signal is well converged to the real signal under a high level of uncertainties and measurement errors.

### 7.2. Fault Detection

Four types of errors are considered, and their effects are examined. First error is named the bias error. A bias error is a constant that is added to the signal. This type of error can be simply detected by a comparison of the signal with other sensor’s measurement. The second type of error is the hard error. In hard error, the sensor result in a constant instead of real value. The third type is the scale error. In the scale error, a constant or a time-varying value is multiplied with the signal. The scale error is more difficult than the bias error. The fourth error is the noise error. In noise error, the actual signal is measured with lower or higher values. To detect this type of error, previous samples of the measured signal are required.

All four described errors are applied on the measured data, and the accuracy of the designed scheme is examined. To see the superiority of designed algorithm better, it is compared with a recurrent fuzzy neural network (RFNN) [36], SVM-NN [37] and neuro-fuzzy (NFLS) [38]. The accuracy of different methods has been shown in Table 1. We see that the suggested method can detect the bias error about 97%, the scaling about 98%, the high noise about 89% and the hard error about 88%. Table 1 verifies that the error detection of the suggested approach is better than other methods. It should be noted that the program is executed 10 times, and the average values are presented.

**Figure 19:**
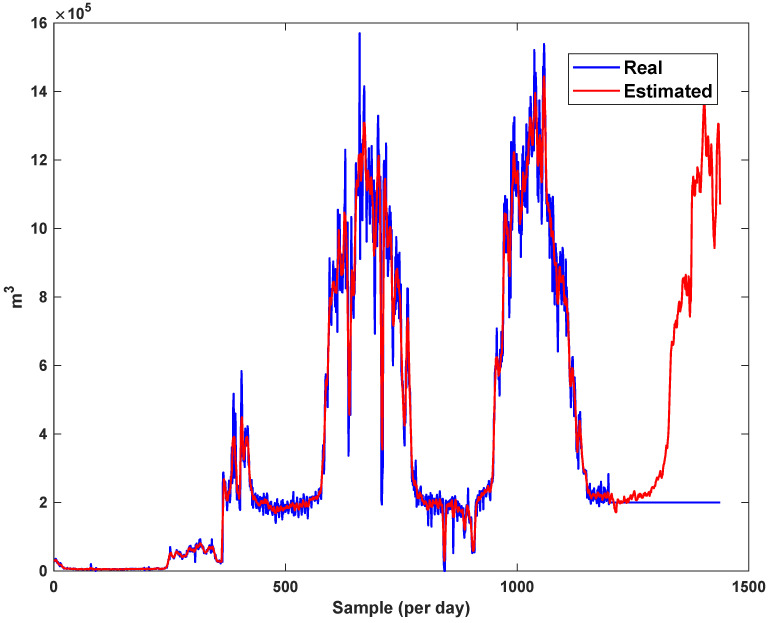
Detection fault 1: Total consumption.

**Figure 20:**
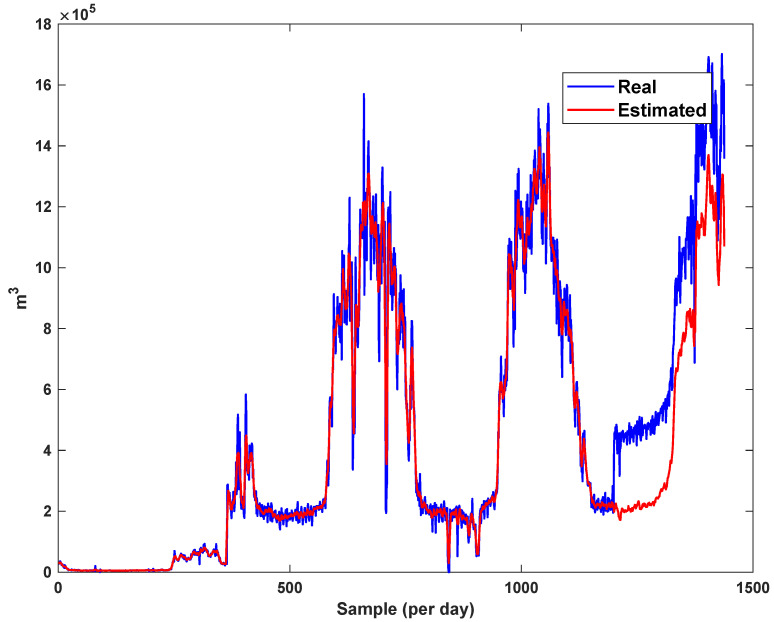
Detection fault 2: Total consumption.

**Figure 21:**
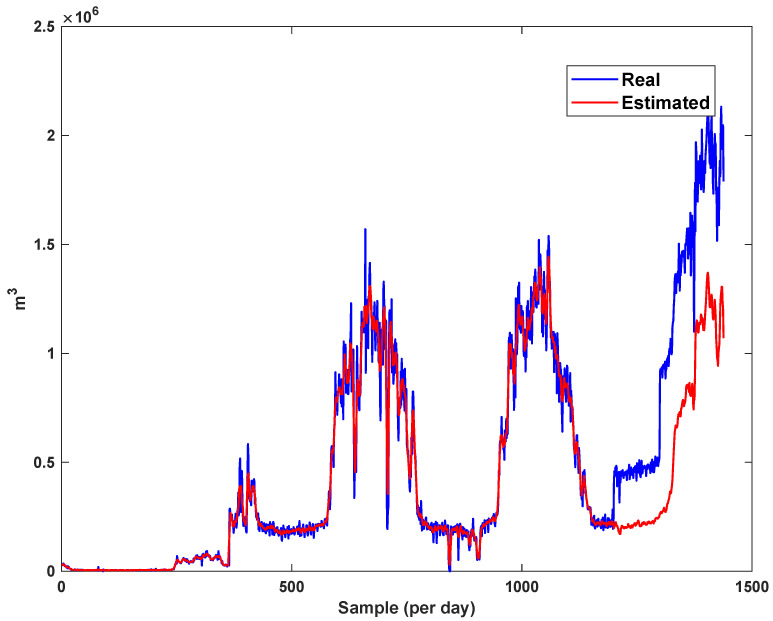
Detection fault 3: Total consumption.

**Figure 22:**
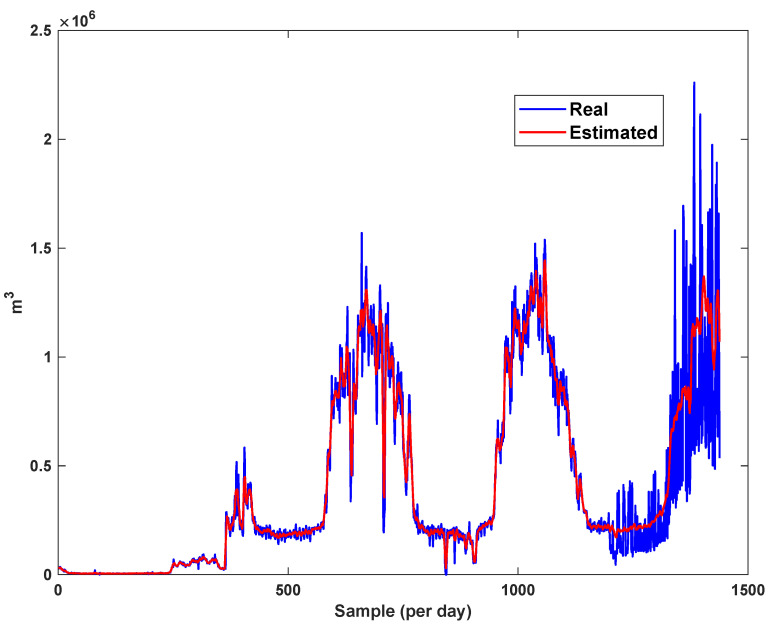
Detection fault 4: Total consumption.

**Figure 23:**
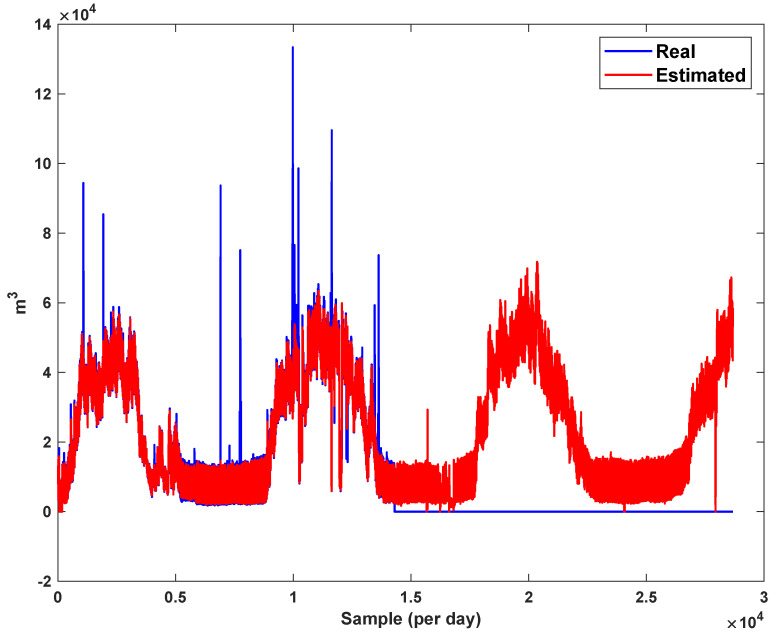
Detection fault 1: Peak of consumption.

**Figure 24:**
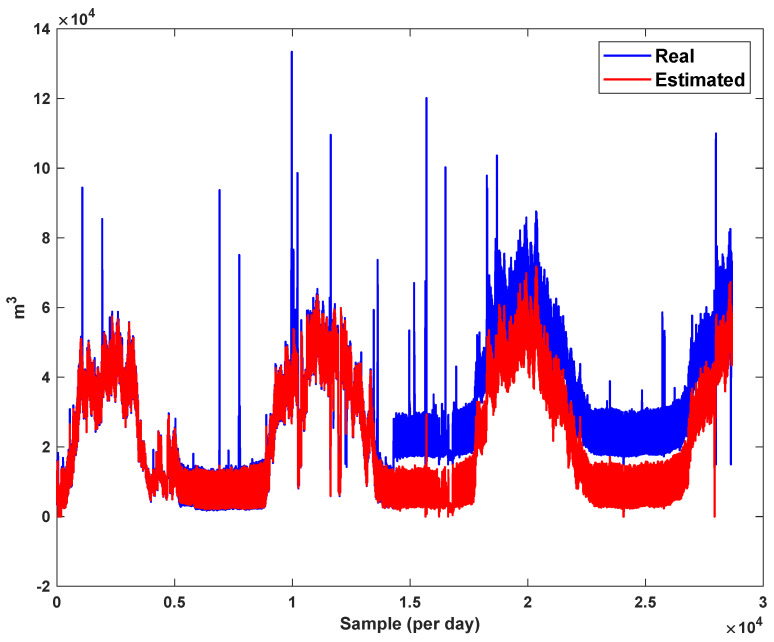
Detection fault 2: Peak of consumption.

**Figure 25:**
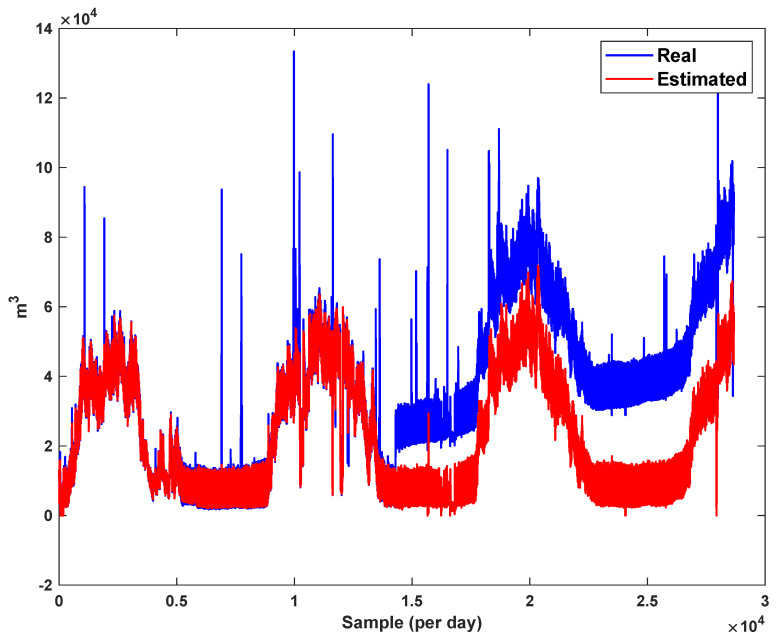
Detection fault 3: Peak of consumption.

**Figure 26:**
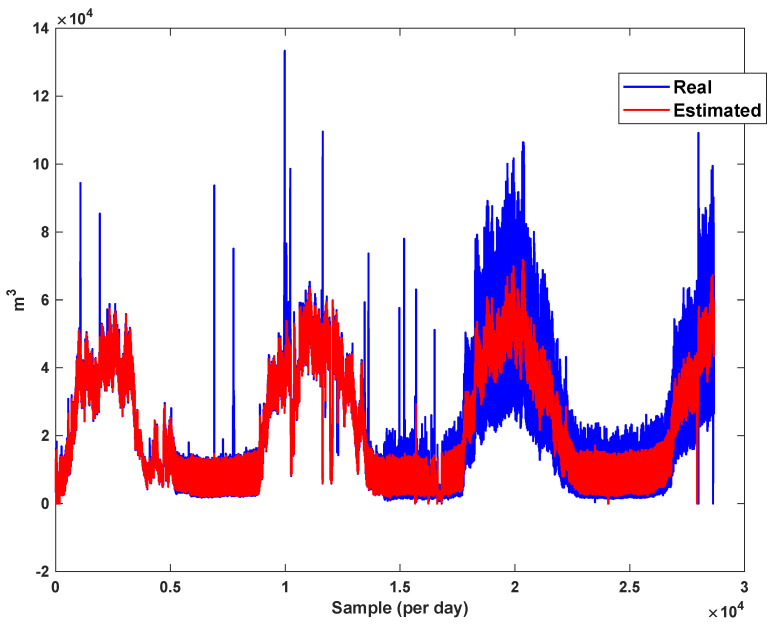
Detection fault 4: Peak of consumption.

The detection of four types of errors for total consumption are shown in Figure 19, Figure 20, Figure 21 and Figure 22. We see that the suggested approach detects all type of errors well. Furthermore, it should be noted that the suggested approach estimates the actual signal well. Similarly, the four types of errors are examined on the peak of consumption data. The results are shown in Figure 23, Figure 24, Figure 25 and Figure 26. It is seen that, in spite of high variation of the actual signal, it is well estimated and predicted. All type of errors are detected with the desired accuracy.

To better show the superiority of non-singleton fuzzification and high-order FLS, a numerical comparison is given. A Gaussian noise is added to the inputs with various variances. The values of the root-mean-square of modeling errors are compared in Table 2. The mean of the results for 10 epoches are given in Table 2. As seen from Table 2, the type-3 FLS by the non-singleton fuzzification results in better accuracy, particularly in the higher noisy condition. It should be noted that all methods are compared in the same condition and same data set.

**Remark** **2.** *It should be noted that the suggested scheme models the output signal, and then based on the a comparison between the model out and the measured signal, it detects the faults. Then, the training data sets should contain no fault. One way to ensure this is measuring of the training data set by multiple sensors. Furthermore, before starting the training process, the abnormal data sets should be filtered and not used in training. Furthermore, it is emphasized that the measurement errors of output signals are handled by the non-singleton fuzzification. In other words, it is assumed that the input data set is not accurate in terms of measuring error. If the training data set includes fault, it is possible that the system could not detect the actual fault.*

**Remark** **3.** *The training process is done in an off-line scheme. Then, the sampling time does not impact the computation cost. Furthermore, in the our-case study system, the speed of changes of gas flow is low, and a fast sampling time is not required. It is obvious that, for fast changing signals, the sampling time should be small enough for better modeling of the signal behavior.*

**Remark** **4.** *The results in Figure 23, Figure 24, Figure 25 and Figure 26 show that the suggested FD scheme gives a good detection performance versus various faults. Furthermore, Table 2 shows a good robustness against disturbances that have been considered as external noises. In Figure 19, Figure 20, Figure 21 and Figure 22, the fault is detected in the total consumption, and we see that the changing of load does not effect the FD performance. We see that increasing the trajectories due to the increasing consumption are not detected as faults, and the suggested FLS learns the consumption pattern well.*

## 8. Conclusions

This paper introduces a new approach for flowmeter fault detection in an experimental project. The actual signals are modeled by the suggested NT3FLS, and the measurement error is well detected, as shown by the comparsion between the predicted signal and measured signal. Four types of errors, such as bias error, scaling error, hard error and noisy measurement error, are considered, and the performance of the suggested scheme is evaluated. First, the data of the case-study system are described, and the modeling accuracy of the suggested approach is examined. It is seen that, in spite of high variation of the actual signals and various errors, the real signals, such as the consumption of three lines and the peak of consumptions, are well estimated by the designed NT3FLS. Second, the accuracy of error detection is examined. The four errors are applied on the total consumption signal and the peak of the consumption signal, and it is shown that the suggested approach detects the error with the desired accuracy. It is shown that the suggested method can detect the bias error about 97%, the scaling about 98%, the high noise about 89% and the hard error about 88%. Comparing the obtained results with some other similar approaches show the superiority of the suggested approach. The main advantages of the suggested approach are: (1) The measurement errors of the recorded historical data are handled by the proposed non-singleton fuzzification. (2) The non-Gaussian noise, which is a prevalent issue in industrial applications, is supported in the suggested scheme. (3) The proposed NT3FLS results in better accuracy in an uncertain environment. 

## Figures and Tables

**Table 1:** Comparison results.

	Method			
**Error Type**	**NT3FLS**	**RFNN [36]**	**SVM-NN [37]**	**NFLS [38]**
Bias	97	90	84	91
Scaling	98	88	85	92
High noise	89	81	80	85
Hard error	88	77	81	80

**Table 2:** Comparison the effect various FLSs.

Noise Variance	FLS			
	Type-1	Type-2	Singleton Type-3	Non-Singleton Type-3
-	0.2208	0.1365	0.0124	0.0117
0.01	0.3481	0.2470	0.1029	0.0921
0.1	0.8427	0.4578	0.3547	0.1804

## Data Availability

The data presented in this study are available on request from Jafar Tavoosi (j.tavoosi@ilam.ac.ir).
